# A variational framework with composite sparse regularization for cryo-electron tomography reconstruction

**DOI:** 10.1093/bioinformatics/btag206

**Published:** 2026-05-26

**Authors:** Chenyun Yu, Zihe Xu, Qiong Zeng, Xiaohua Wan, Haythem El-Messiry, Fa Zhang, Renmin Han

**Affiliations:** Frontiers Science Center for Nonlinear Expectations (Ministry of Education), Research Center for Mathematics and Interdisciplinary Sciences, Qilu Hospital (Qingdao), Cheeloo College of Medicine, Shandong University, Qingdao, 266237, China; College of Medical Information and Engineering, Ningxia Medical University, Yinchuan, 750004, China; Frontiers Science Center for Nonlinear Expectations (Ministry of Education), Research Center for Mathematics and Interdisciplinary Sciences, Qilu Hospital (Qingdao), Cheeloo College of Medicine, Shandong University, Qingdao, 266237, China; School of Computer Science and Technology, Shandong University, Qingdao, 266237, China; School of Medical Technology, Beijing Institute of Technology, Beijing, 100081, China; Canadian University Dubai, Dubai, 117781, United Arab Emirates; School of Medical Technology, Beijing Institute of Technology, Beijing, 100081, China; Frontiers Science Center for Nonlinear Expectations (Ministry of Education), Research Center for Mathematics and Interdisciplinary Sciences, Qilu Hospital (Qingdao), Cheeloo College of Medicine, Shandong University, Qingdao, 266237, China; College of Medical Information and Engineering, Ningxia Medical University, Yinchuan, 750004, China

## Abstract

**Motivation:**

Cryogenic electron tomography (cryo-ET) enables *in situ* visualization of macromolecular and cellular structures from tilt-series projections. Reconstruction quality is often compromised by extremely low signal-to-noise ratio (SNR) and vignetting artifacts arising from detector truncation under constrained acquisition geometries. In practice, existing methods frequently struggle to balance noise robustness, computational efficiency, and stability under these conditions.

**Results:**

We propose a robust, scalable, and parallelizable variational reconstruction framework that integrates a geometrically consistent data fidelity term with an implicit boundary-handling mechanism to mitigate truncation-induced artifacts without volume padding. A composite sparse regularizer integrating anisotropic total variation and curvelet-domain sparsity is employed to preserve structural boundaries and multiscale directional features. The resulting optimization problem is efficiently solved using the primal-dual hybrid gradient (PDHG) algorithm without nested inner iterations, for which we provide rigorous theoretical guarantees of stability and convergence. Experiments on simulated and experimental cryo-ET datasets demonstrate substantial noise suppression and contrast enhancement while preserving fine structural details under realistic, severely noise-limited and truncated acquisition conditions. These improvements lead to enhanced interpretability and facilitate downstream structural analysis, while achieving significantly reduced runtime compared to existing methods at comparable reconstruction quality.

**Availability and implementation:**

Our code available at https://github.com/icthrm/CSRT. The real datasets used in this study are publicly available from EMPIAR and the Caltech Electron Tomography Database.

## 1 Introduction

Cryogenic electron tomography (cryo-ET) enables three-dimensional (3D) visualization of pleomorphic and heterogeneous biological specimens in a near-native state, providing direct access to *in situ* cellular architecture from a tilt series of two-dimensional (2D) transmission projections (Han and Xu 2024). Achieving high-fidelity reconstructions, however, is severely impeded by fundamental physical and instrumental constraints. The electron dose must be kept extremely low to mitigate radiation damage, resulting in projection images with critically low signal-to-noise ratio (SNR). In addition, the accessible tilt range is restricted by microscope geometry, giving rise to the missing wedge problem; for subtomogram analysis, alignment and averaging can partially account for the missing wedge during similarity estimation and refinement, reducing the associated bias (Bartesaghi *et al*. 2008, Han *et al*. 2021). Beyond limited-angle sampling, cryo-ET reconstructions can also suffer from systematic bias due to the limited detector, often referred to as the long-object problem. Specifically, when the specimen extends beyond the reconstruction region of interest (ROI), the measured projections include contributions from outside the ROI that are absent from the forward model, leading to peripheral intensity fall-off and cupping or vignetting artifacts. Limited-detector compensation has been proposed to mitigate this mismatch and suppress vignetting (Xu *et al*. 2010).

A broad spectrum of reconstruction algorithms has been used in cryo-ET, with many microscopy pipelines relying on classical methods due to their simplicity and throughput. Direct approaches such as filtered back-projection (FBP) and weighted back-projection (WBP) are computationally efficient and therefore remain common in routine processing Kak and Slaney (2001). Algebraic iterative methods, including the simultaneous iterative reconstruction technique (SIRT) (Gilbert 1972) and the simultaneous algebraic reconstruction technique (SART), improve data agreement through repeated forward-backward updates and are often favored for their robustness relative to direct back-projection under noisy and limited-angle conditions; under appropriate weighting, such iterations can be related to weighted least-squares (WLS) objectives (Van Der Sluis and Van Der Vorst 1990, Gregor and Fessler 2015). These algorithms are implemented and widely accessed through established software ecosystems for cryo-ET processing, including IMOD (Kremer *et al*. 1996), Tomo3D (Agulleiro and Fernandez 2011, 2015), EMAN2 (Tang *et al*. 2007), AuTom (Han *et al*. 2017, 2019), and acceleration-oriented toolkits such as TiltRec (Jiao *et al*. 2025) (see also (Pyle and Zanetti 2021)). Nevertheless, under low dose and missing-wedge sampling, classical reconstructions can exhibit substantial noise amplification, contrast loss, and iterative schemes may still rely largely on implicit regularization, for example through early stopping, reflecting the ill-posed nature of electron tomographic inversion (Fernandez 2012, Turoňová *et al*. 2015).

These limitations have motivated methods that incorporate stronger priors and better exploit the structure of cryo-ET data, most prominently through variational reconstruction frameworks and, more recently, deep learning. A statistically grounded model based iterative reconstruction (MBIR) framework was introduced in (Yan *et al*. 2019). It improves contrast by modeling the measurement process and incorporating object priors, but it is often computationally expensive because it relies on iterative MAP optimization and costly likelihood and prior terms (Sabne *et al*. 2017). A higher-order total variation (TV)-regularized reconstruction model was formulated in (Böhning *et al*. 2022) and solved using a primal-dual hybrid gradient (PDHG) algorithm (Chambolle and Pock 2011), while vignetting artifacts were reported that may require cropping in downstream subtomogram analysis. A GPU-accelerated proximal framework based on a linearized alternating direction method of multipliers (LADMM) was proposed in (Ramirez *et al*. 2024), but it incurs costly inner iterations, repeatedly invokes SART within the splitting updates, and pads the reconstruction volume to handle limited detector coverage, increasing memory and computation. Meanwhile, deep learning has become a complementary approach for cryo-ET denoising and missing-wedge compensation, spanning learning-based reconstruction and self-supervised fitting to measured data (Yang *et al*. 2021, Liu *et al*. 2022, Wiedemann and Heckel 2024). However, such data-driven methods can raise concerns about scientific trustworthiness and reproducibility. When probing previously unknown *in situ* architectures, strict data fidelity is critical, since hallucinated features may mislead biological interpretation.

In this work, we propose a variational reconstruction framework to address these challenges via a regularized optimization model. First, we formulate a geometrically consistent WLS data fidelity term with an integrated limited-detector compensation scheme that suppresses vignetting artifacts without padding the reconstruction volume. Second, leveraging the modularity of proximal splitting, we implement a composite sparse regularization that combines anisotropic TV (ATV) with curvelet-domain sparsity, balancing boundary preservation with the recovery of multiscale directional features. Third, we solve the resulting non-smooth problem using an efficient PDHG algorithm, which decomposes the optimization into simple element-wise updates and avoids the costly nested inner iterations typical of LADMM-based methods, yielding improved scalability and higher computational efficiency among methods with comparable reconstruction quality. Moreover, we provide rigorous stability and convergence guarantees for the proposed algorithm, strengthening the reliability of the reconstructions for biological analysis. We validate the framework on simulated data and multiple experimental cryo-ET datasets using visual inspection, complementary quantitative metrics, and downstream task performance, demonstrating substantial noise suppression and contrast enhancement while preserving fine structural details.

## 2 Proposed method

This section presents the proposed variational framework for cryo-ET reconstruction, including its formulation, theoretical properties, and numerical solution. The reconstruction problem is introduced together with the associated notation, followed by the definition of an objective function that combines a truncation-aware data fidelity term and a composite sparse regularizer. Subsequent subsections detail the derivation of the weighted-norm fidelity model, the design of the regularization terms, and the PDHG-based optimization algorithm with stability and convergence guarantees.

### 2.1 Problem formulation

Let $u\in R^{N}$ denote the unknown 3D volume (vectorized) to be reconstructed from a tilt series, and let $b\in R^{M}$ denote the measured projection data after standard preprocessing (e.g., alignment and optional binning). The forward projection operator is written as $A:R^{N}\to R^{M}$, with adjoint (backprojection) $A^{T}$. We use a diagonal weighting matrix $R\in R^{M\times M}$ and the corresponding weighted norm.


$$∥x{∥}_{R}^{2}≜x^{T}\mathrm{Rx}.$$

We estimate u in a variational manner by minimizing a sum of a data fidelity term and a regularization term:


$$\underset{u\in R^{N}}{\min}f(u)+\Omega (u).$$

The fidelity term f(u) is constructed to be geometrically consistent under the limited-detector setting by incorporating truncation compensation, aiming to suppress vignetting artifacts without padding the reconstruction volume. To regularize the reconstruction while retaining both boundary information and multiscale directional structures, we use a composite sparse regularizer that combines complementary priors rather than relying on a single penalty term.


$$\Omega (u)=\lambda_{1}\Omega_{1}(u)+\lambda_{2}\Omega_{2}(u),\;\lambda_{1},\lambda_{2}>0,$$

Where in this work $\Omega_{1}$ corresponds to ATV and $\Omega_{2}$ promotes sparsity in the curvelet domain.

In the following, we first derive the weighted-norm data fidelity term together with the truncation compensation mechanism, and then present the composite regularization operators and the PDHG solver used to optimize the resulting non-smooth objective.

### 2.2 Weighted-norm data fidelity

The foundation of our reconstruction model is a WLS data fidelity term. In standard algebraic reconstruction, the iterative updates are typically expressed as


$$u^{k+1}=u^{k}-CA^{T}R\left(Au^{k}-b\right),$$

Where R and C are diagonal weighting matrices containing the inverse row and column sums of the system matrix A, respectively,


$$r_{ii}=\frac{1}{\sum_{j=1}^{N}a_{ij}},\;c_{jj}=\frac{1}{\sum_{i=1}^{M}a_{ij}}.$$

Here, we assume that the system matrix entries are nonnegative, i.e., $a_{ij}\geq 0$, as they represent projection weights in tomographic imaging. Moreover, for the rows and columns involved in the reconstruction, the corresponding sums are strictly positive, that is, $\sum_{j=1}^{N}a_{ij}>0$ and $\sum_{i=1}^{M}a_{ij}>0$. This iteration implicitly minimizes the weighted objective function $f(u)=\frac{1}{2}∥\mathrm{Au}-b{∥}_{R}^{2}$ (Gregor and Fessler 2015).

**Figure 1:**
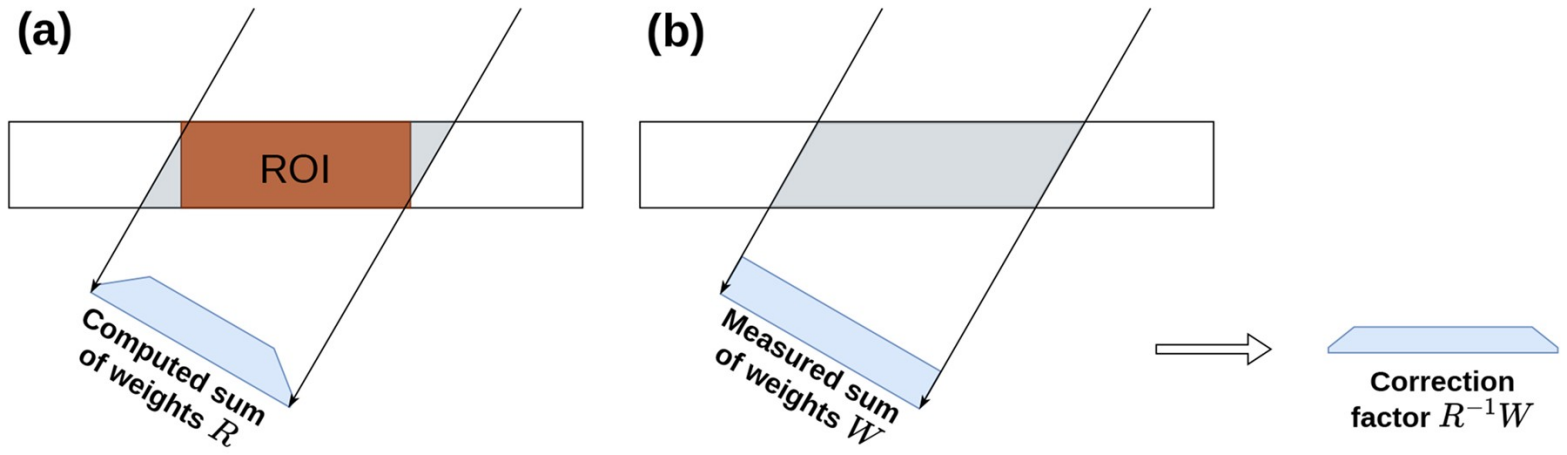
Illustration of the limited detector problem. (a) The gray area highlights regions of the specimen outside the defined reconstruction volume (ROI) that still contribute to the measured projection. The blue trapezoid represents the computed sum of weights within the ROI. (b) The blue rectangle represents the theoretical sum of weights corresponding to the full ray path through the specimen. The derived factor is applied for correction.

However, a fundamental physical mismatch arises in cryo-ET due to the limited detector problem. As illustrated in Fig. 1, the acquired projection data b integrates information from the entire physical specimen, which often extends beyond the field of view. In contrast, the forward projection Au accounts solely for voxels within the defined reconstruction volume. Direct subtraction of these two terms leads to severe vignetting and cupping artifacts because b is physically scaled by the full object geometry, while Au is scaled only by the ROI.

To resolve this inconsistency, we directly integrate the weight correction scheme in (Xu *et al*. 2010) into our variational framework. Let W denote the theoretical diagonal weighting matrix corresponding to the ray path lengths through the full specimen geometry. A physically consistent residual term, which compares the average density per unit length, is formulated as


(1) residual=RAu−Wb.

Here, RAu represents the normalized projection of the reconstruction volume, and Wb represents the normalized acquired data. To incorporate this correction into our solver, we factor out the matrix R:


(2) $$\mathrm{RAu}-\mathrm{Wb}=R\left(\mathrm{Au}-R^{-1}\mathrm{Wb}\right).$$

This derivation reveals that limited detector compensation is mathematically equivalent to replacing the raw projection data b with a corrected vector


(3) $$\tilde{b}=R^{-1}\mathrm{Wb}.$$

Consequently, our final data fidelity term is defined as


(4) $$f(u)=\frac{1}{2}∥\mathrm{Au}-\tilde{b}{∥}_{R}^{2}.$$

In practice, this correction is implemented as a pre-processing step. Since the exact geometry of the exterior object is unknown, the elements of W are approximated analytically by modeling the full specimen as a slab of uniform thickness *d*. The path length for a ray at tilt angle θ is estimated as L=d/ cos(θ), and the weights are set as the inverse of these lengths. By solving for $\tilde{b}$ instead of b, severe vignetting artifacts arising from the limited detector coverage are effectively eliminated.

### 2.3 Composite regularization

To obtain noise-robust yet structurally faithful reconstructions in cryo-ET, we use a composite regularization term that combines two complementary priors. We adopt ATV to preserve sharp boundaries and enforce local smoothness, and we further impose curvelet-domain sparsity to capture multiscale directional features. Together, these priors reduce noise while better maintaining fine biological structures than a single penalty term.

### 2.4 Anisotropic total variation

TV regularization has been widely adopted in electron tomogram reconstruction primarily for its robust denoising capabilities (Goris *et al*. 2012). Biologically, cryo-ET volumes typically consist of macromolecular complexes and organelles embedded in vitreous ice. The density transitions between these biological structures and the background solvent are ideally sharp, approximating a piecewise-constant intensity model (Deng *et al*. 2016).

Mathematically, TV minimization promotes sparsity in the gradient domain. In the context of cryo-ET, this property is particularly effective at suppressing the severe high-frequency noise inherent to low-dose imaging while preserving the distinct boundaries of membranes and protein shells. By enforcing local smoothness within homogeneous regions, TV enhances the SNR and facilitates the visual delineation of structural features, which is critical for downstream segmentation tasks.

For a discrete 3D volume $u\in R^{N}$, the Isotropic TV (ITV) is often theoretically preferred for its rotational invariance:


(5) $$∥\nabla u{∥}_{2,1}=\sum_{i=1}^{N}\sqrt{{\left(D_{x}u\right)}_{i}^{2}+{\left(D_{y}u\right)}_{i}^{2}+{\left(D_{z}u\right)}_{i}^{2}}.$$

However, the coupling of derivatives under the square root in ITV precludes a closed-form solution for its proximal operator, typically necessitating computationally expensive iterative sub-solvers. To ensure computational efficiency for large-scale tomograms, we adopt the $\ell_{1}$-based ATV, defined by:


(6) $$∥\nabla u{∥}_{1}=\sum_{i=1}^{N}\left(\left|{\left(D_{x}u\right)}_{i}|+|{\left(D_{y}u\right)}_{i}|+|{\left(D_{z}u\right)}_{i}\right|\right),$$

Where $D_{x},D_{y},D_{z}$ represent the linear forward finite difference operators along the three spatial dimensions. The ATV formulation decouples the problem across spatial dimensions, yielding a fully separable proximal operator that allows the regularization update to be computed efficiently via simple component-wise soft-thresholding operations, thus avoiding the need for nested inner iterations. Consequently, we set $\Omega_{1}(u)=∥\nabla u{∥}_{1}$.

### 2.5 Curvelet-Domain sparsity

Standard TV regularization is widely used for preserving sharp boundaries (Rudin *et al*. 1992), but its axis-aligned gradient penalty can be insufficient for modeling curved and multiscale textures and may lead to “staircasing” artifacts (Böhning *et al*. 2022). Motivated by the directional and multiscale nature of cryo-ET structures, we introduce a curvelet sparsity prior as the second regularization term $\Omega_{2}(u)$. Curvelets provide an optimally sparse representation for images with $C^{2}$-singularities such as smooth curves and wavefronts (Candès *et al*. 2006), and have been successfully applied in tomographic inverse problems, including sparse-view settings and hybrid formulations with TV (Yazdanpanah and Regentova 2016, Durand *et al*. 2017).

Let C denote the forward 3D fast discrete curvelet transform (FDCT) operator described in (Ying *et al*. 2005, Candès *et al*. 2006). We define $\Omega_{2}(u)$ as the $\ell_{1}$-norm of the curvelet coefficients:


(7) $$\Omega_{2}(u)=∥Cu{∥}_{1}.$$

In our implementation, C is computed using the wrapping-based FDCT in the CurveLab toolbox, which offers favorable computational efficiency and near-isometry. The $\ell_{1}$ penalty promotes sparsity in the curvelet domain by encouraging most coefficients to be small while retaining a limited number of significant coefficients that encode dominant multiscale directional structures.

### 2.6 Numerical optimization via the PDHG algorithm

Substituting the weighted data-fidelity term and the composite sparsity regularizers into the generic variational model yields the unconstrained problem


(8) $$\underset{u\in R^{N}}{\min}\;\{\frac{1}{2}∥\mathrm{Au}-\tilde{b}{∥}_{R}^{2}+\lambda_{1}∥\nabla u{∥}_{1}+\lambda_{2}∥Cu{∥}_{1}\}.$$

We solve this non-smooth problem using the primal–dual hybrid gradient (PDHG) algorithm (Chambolle and Pock 2011) by casting it into the saddle-point form


$$\underset{u}{\min}\underset{v}{\max}\;\langle \mathrm{Ku},v\rangle -F^{*}(v)+G(u),\;G(u)\equiv 0.$$

The stacked linear operator is


$$\begin{array}{c} K=\left[\begin{array}{c} R^{1/2}A\\ \nabla \\ C \end{array}\right],\;K^{*}=\left[\begin{array}{ccc} A^{T}R^{1/2} & \nabla^{*} & C^{*} \end{array}\right], \end{array}$$

Where $\nabla^{*}$ and $C^{*}$ denote the adjoints of the finite-difference and curvelet operators, respectively. Let $v={\left(v_{A},v_{\nabla },v_{C}\right)}^{T},$ we define the block-separable function


$$F(v)=\frac{1}{2}∥v_{A}-R^{1/2}\tilde{b}{∥}_{2}^{2}+\lambda_{1}∥v_{\nabla }{∥}_{1}+\lambda_{2}∥v_{C}{∥}_{1}.$$

PDHG alternates a proximal ascent step on the dual variable and a gradient descent step on the primal variable:


(9) $$v^{k+1}={\mathrm{prox}}_{\sigma F^{*}}(v^{k}+\sigma K{\bar{u}}^{k}),$$

(10) $$u^{k+1}=u^{k}-\tau K^{*}v^{k+1},$$

(11) $${\bar{u}}^{k+1}=u^{k+1}+\theta (u^{k+1}-u^{k}).$$

Next, we derive closed-form updates for each dual block $\left(v_{A},v_{\nabla },v_{C}\right)$.

### 2.7 Data fidelity update

The conjugate of the shifted quadratic data term $F_{A}$ is $F_{A}^{*}(v_{A})=\frac{1}{2}∥v_{A}{∥}_{2}^{2}+\langle v_{A},R^{1/2}\tilde{b}\rangle$. Its proximal operator admits a closed-form linear solution:


(12) $${\mathrm{prox}}_{\sigma F_{A}^{*}}(w)=\frac{w-\sigma R^{1/2}\tilde{b}}{1+\sigma }.$$

Applying this to the general dual update step as shown in Eq. (9), the update for $v_{A}$ becomes:


(13) $$v_{A}^{k+1}=\frac{v_{A}^{k}+\sigma R^{1/2}(A{\bar{u}}^{k}-\tilde{b})}{1+\sigma }.$$

Direct evaluation of Eq. (13) requires the computationally expensive matrix square root $R^{1/2}$. To circumvent this, we introduce the scaled dual variable ${\tilde{v}}_{A}=R^{1/2}v_{A}$. Multiplying Eq. (13) by $R^{1/2}$ yields an efficient update rule that relies solely on the original weight matrix R:


(14) $${\tilde{v}}_{A}^{k+1}=\frac{{\tilde{v}}_{A}^{k}+\sigma R(A{\bar{u}}^{k}-\tilde{b})}{1+\sigma }.$$

Consequently, the contribution to the primal update step (10) simplifies to $A^{T}R^{1/2}v_{A}^{k+1}=A^{T}{\tilde{v}}_{A}^{k+1}$.

### 2.8 Regularization update

For the regularization terms, the convex conjugates are indicator functions of $\ell_{\infty }$-balls, i.e., $F_{\nabla }^{*}=\delta_{B_{\lambda_{1}}^{\infty }}$ and $F_{C}^{*}=\delta_{B_{\lambda_{2}}^{\infty }}$. Their proximal operators correspond to simple element-wise projections:


(15) $${\mathrm{prox}}_{\sigma \delta_{B_{\lambda }^{\infty }}}(w)=\Pi_{\lambda }(w)=\text{sign}(w)⊙\min(|w|,\lambda ).$$

Thus, the dual variables $v_{\nabla }$ and $v_{C}$ are updated by projecting their respective arguments onto the balls of radius $\lambda_{1}$ and $\lambda_{2}$.

Substituting these specific operators into the iterative scheme yields the implementation detailed in Algorithm 1.

Algorithm 1 PDHG Algorithm for the Composite Regularized Model (8)

1: Operators $A,A^{T},\nabla ,\nabla^{*},C,C^{*}$; Weight matrix R; Corrected data $\tilde{b}$; Parameters $\lambda_{1},\lambda_{2},\tau ,\sigma ,\theta$.

2: Initialize primal variables $u^{0}={\bar{u}}^{0}=0$ and dual variables ${\tilde{v}}_{A}^{0},v_{\nabla }^{0},v_{C}^{0}$ to 0.

3: **for**  $k=0,1,\ldots ,K_{\max}-1$  **do** 

4:   *//Dual Update: Data Fidelity*

5:

$${\tilde{v}}_{A}^{k+1}\leftarrow \frac{{\tilde{v}}_{A}^{k}+\sigma R(A{\bar{u}}^{k}-\tilde{b})}{1+\sigma }$$

6:   *//Dual Update: Regularization (*$\ell_{\infty }$*-ball projections)*

7:   $v_{\nabla }^{k+1}\leftarrow \text{sign}(w_{\nabla })⊙\min(|w_{\nabla }|,\lambda_{1})$, where $w_{\nabla }=v_{\nabla }^{k}+\sigma \nabla {\bar{u}}^{k}$

8:   $v_{C}^{k+1}\leftarrow \text{sign}(w_{C})⊙\min(|w_{C}|,\lambda_{2})$, where $w_{C}=v_{C}^{k}+\sigma C{\bar{u}}^{k}$

9:   *//Primal Update*

10:   $u^{k+1}\leftarrow u^{k}-\tau \left(A^{T}{\tilde{v}}_{A}^{k+1}+\nabla^{*}v_{\nabla }^{k+1}+C^{*}v_{C}^{k+1}\right)$

11:   *//Extrapolation*

12:   ${\bar{u}}^{k+1}\leftarrow u^{k+1}+\theta (u^{k+1}-u^{k})$

13: **end for** 

14: **return**  $u^{k+1}$

### 2.9 Convergence analysis

We provide theoretical guarantees on the stability and convergence of the proposed PDHG solver under standard assumptions. Due to space limitations, the complete analysis, including supporting lemmas and proofs, is provided in Supplementary Section S1, available as supplementary data at *Bioinformatics* online.

## 3 Results

This section evaluates the proposed framework on simulated and experimental cryo-ET data. We report qualitative comparisons, leave-one-out FRC for measurement consistency, template matching for particle detection, and subtomogram averaging with FSC-based resolution on the simulated dataset.

### 3.1 Datasets and experimental setup

To evaluate the proposed method, we conducted experiments on one simulated dataset and four experimental cryo-ET datasets (EMPIAR-10643, EMPIAR-10499, EMPIAR-10110, and Nitrosop3). Dataset statistics and detailed acquisition parameters for each dataset are provided in Supplementary Section S2, available as supplementary data at *Bioinformatics* online. For all experimental datasets, the raw projections were spatially binned by a factor of 4 prior to reconstruction, and all tilt series were aligned using Markerauto2 Xu *et al*. (2024).

As comparison methods, we considered WBP, SIRT, MBIR, and LADMM. WBP and SIRT reconstructions were performed using Tomo3D, whose SIRT implementation also includes long object compensation. The parameters of the comparison methods were selected based on overall visual quality, noise suppression, and downstream task performance. In particular, the Hamming weighting filter frequency for WBP was set to 0.0, and SIRT was run for 10 iterations in all comparative experiments. Additional parameter sensitivity comparisons for WBP and SIRT are provided in Supplementary Section S3, available as supplementary data at *Bioinformatics* online. MBIR was configured following the parameter setting procedure described in Yan *et al*. (2019) and its supplementary material. For LADMM and our method, the parameters were determined by grid search, and the final setting was selected based on visual quality and downstream task performance. Moreover, once determined for a given dataset, the parameter settings of each method were used unchanged across all experiments involving that dataset. All experiments were conducted on a computing cluster node equipped with an Intel Xeon Platinum 8260 CPU and an NVIDIA A100 GPU with 40 GB memory.

### 3.2 Visual quality assessment

**Figure 2:**
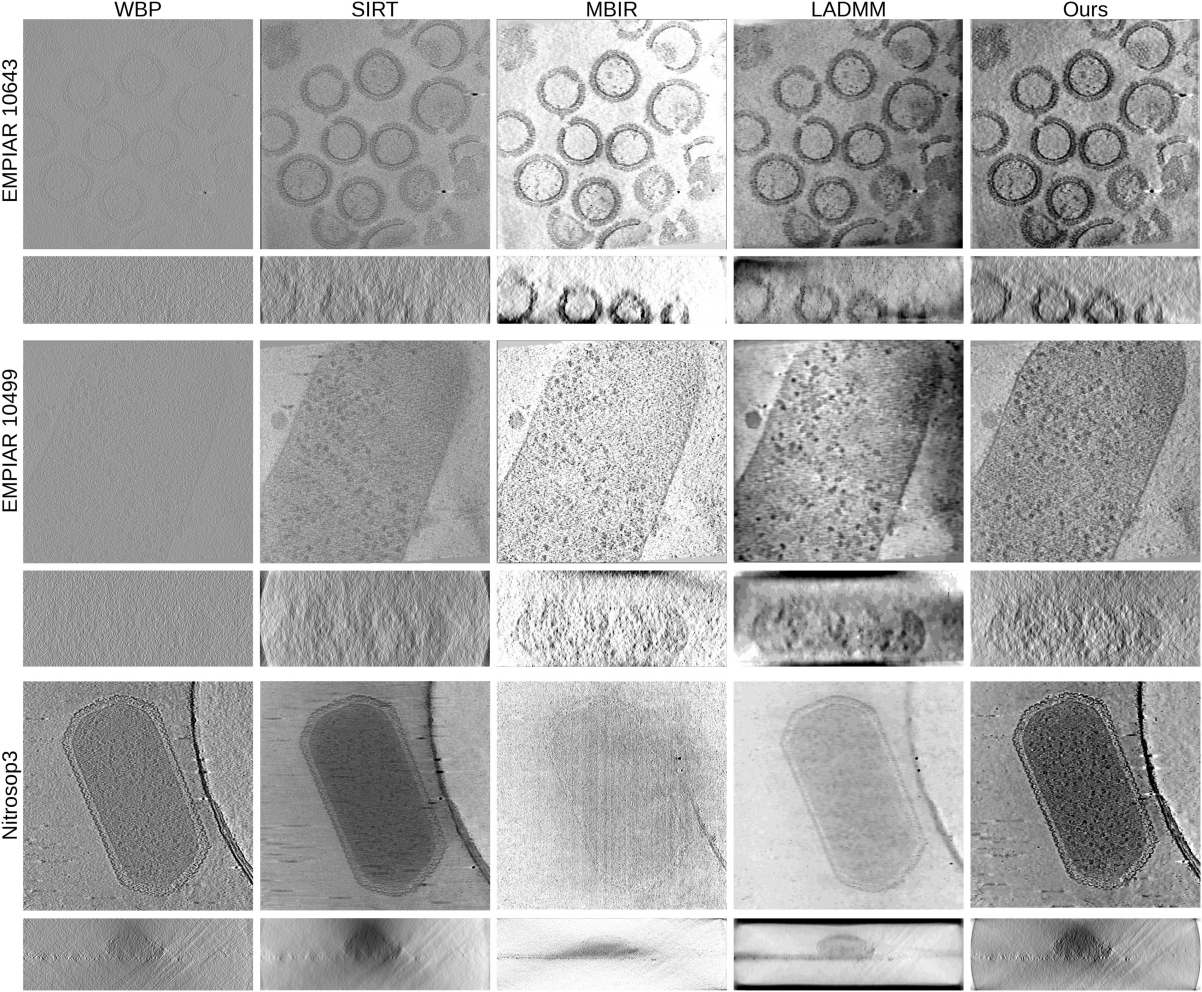
Visual comparison of XY and XZ slices from reconstruction results across real cryo-ET datasets. The columns correspond to different reconstruction methods (WBP, SIRT, MBIR, LADMM, and Ours), and the rows correspond to different biological specimens (EMPIAR-10643, EMPIAR-10499, and Nitrosop3).

The XY slices in Fig. 2 highlight differences in structural visibility, contrast, and detail preservation among the reconstruction methods. WBP suffers from a low SNR, so only the approximate boundaries and the presence of particle like features can be recognized. SIRT makes boundaries and particle like features easier to distinguish than in WBP, but the contrast remains low and the noise level is still relatively high. MBIR performs well on EMPIAR-10643 and EMPIAR-10499, where discrete structures such as viral capsids and ribosomes are recovered with clearer interiors and more coherent edges; however, it is less favorable on the macroscopic Nitrosop3 dataset, where the reconstruction appears low in contrast and several cellular components remain poorly separated. LADMM suppresses background fluctuations, but it tends to over smooth the volume and weaken structural contrast, resulting in ambiguous cell envelope boundaries and a flattened intracellular appearance, particularly in Nitrosop3 where texture cues become less informative. In contrast, our method consistently enhances contrast and sharpness across all three datasets, improving the separation between structures and background while better preserving boundaries and fine structural details.

The XZ slices in Fig. 2 provide a more direct view of the limited detector problem, where the corresponding vignetting artifacts are more evident. Among the compared methods, SIRT in Tomo3D and our method are the two approaches that explicitly incorporate long object compensation. As shown in the XZ views, both methods effectively alleviate the vignetting artifacts caused by the limited detector problem, consistent with the behavior illustrated in Xu *et al*. (2010). In particular, the strong intensity imbalance near peripheral regions is substantially reduced, indicating that long object compensation is effective for regions whose tilted projections remain within the detector field of view. Some residual local intensity differences can still be observed in boundary areas with extremely limited projection support, but these are confined to regions where parts of the object extend beyond the recorded projection area at certain tilt angles. Overall, the XZ results further support that our method handles the limited detector problem well.

**Figure 3:**
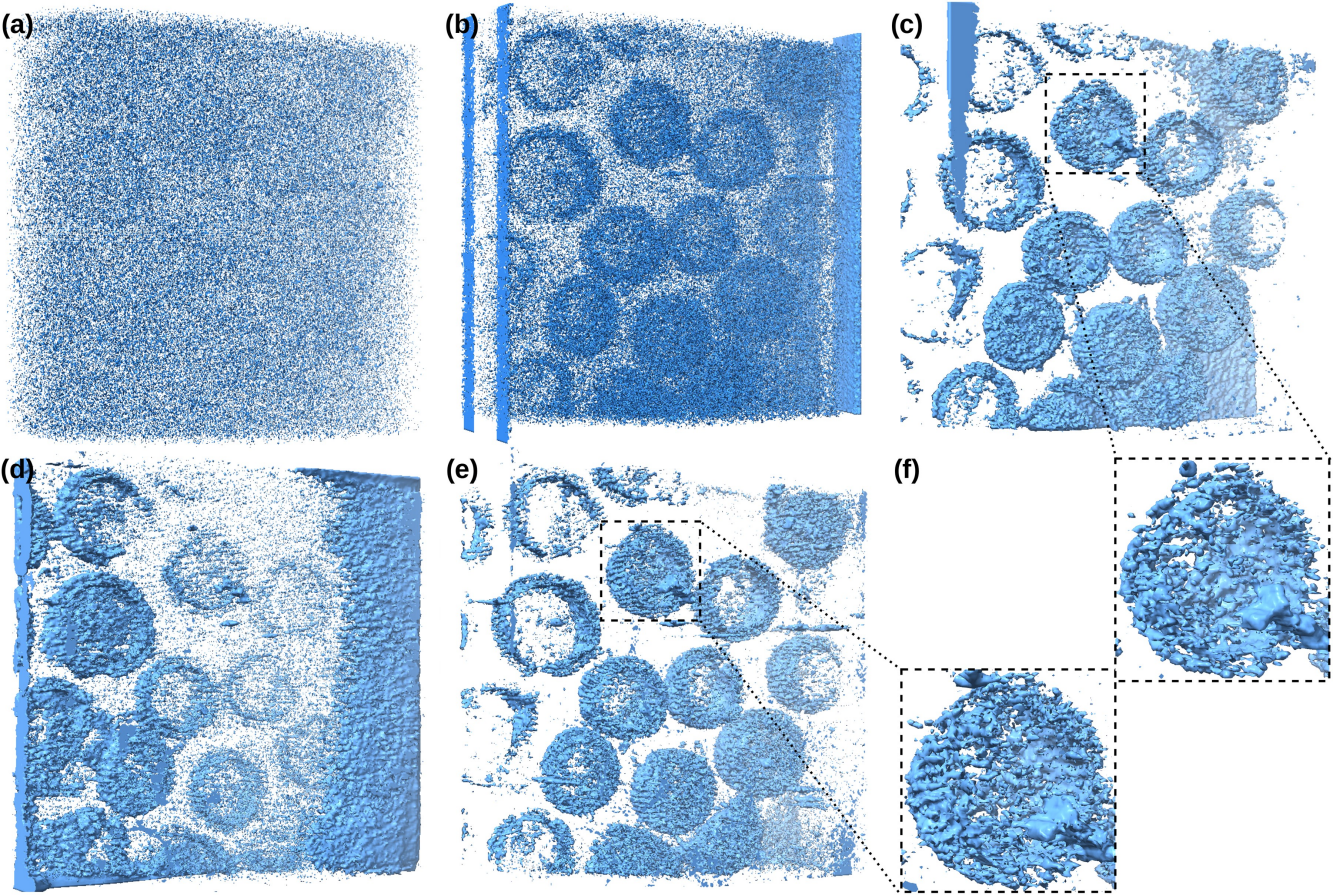
3D isosurface rendering comparison of the HIV-1 dataset (EMPIAR-10643) visualized using UCSF ChimeraX. (a) WBP. (b) SIRT. (c) MBIR. (d) LADMM. (e) Our proposed method. (f) Detailed close-up comparison between MBIR and our method. The dashed boxes in (c) and (e) indicate the zoomed-in regions displayed in (f), where the top crop corresponds to the MBIR result and the bottom crop corresponds to our method.

Complementing the 2D slice analysis, Fig. 3 presents 3D isosurface renderings of the HIV-1 dataset (EMPIAR-10643) visualized in ChimeraX Goddard *et al*. (2018) to assess structural fidelity. WBP is dominated by noise, producing a heavily fragmented volume in which the capsid particles are barely distinguishable. SIRT recovers the approximate particle shapes more clearly than WBP, but the reconstructed surfaces remain noisy and irregular, which still limits the clarity of the capsid shells. Regularized methods reduce these artifacts and recover overall particle shapes, but differences remain in structural completeness and surface texture. LADMM, although strongly denoised, shows structural loss, including incomplete shells and overly smooth surfaces that remove meaningful geometric variation. MBIR yields a clean visualization close to our result, yet Fig. 3f shows that it smooths intrinsic surface granularity and slightly reduces local detail on the capsid shell. By contrast, our method maintains a continuous and denoised surface while retaining more shell texture and local geometric variation, indicating a better balance between noise suppression and preservation of biologically relevant detail.

### 3.3 Fidelity assessment via leave-one-out FRC

We assessed reconstruction fidelity using leave-one-out Fourier ring correlation (FRC) Cardone *et al*. (2005), which evaluates how well a reconstructed tomogram predicts an unseen measurement. For each aligned projection *X* at a given tilt angle, we excluded it from the tilt series, reconstructed a tomogram from the remaining projections, and re-projected the tomogram at the omitted angle to obtain a prediction $\tilde{X}$. We then computed the FRC between *X* and $\tilde{X}$, where higher correlations over spatial frequencies indicate better measurement consistency and information restoration. LADMM was not included because the released implementation does not support leaving out individual projections.

**Figure 4:**
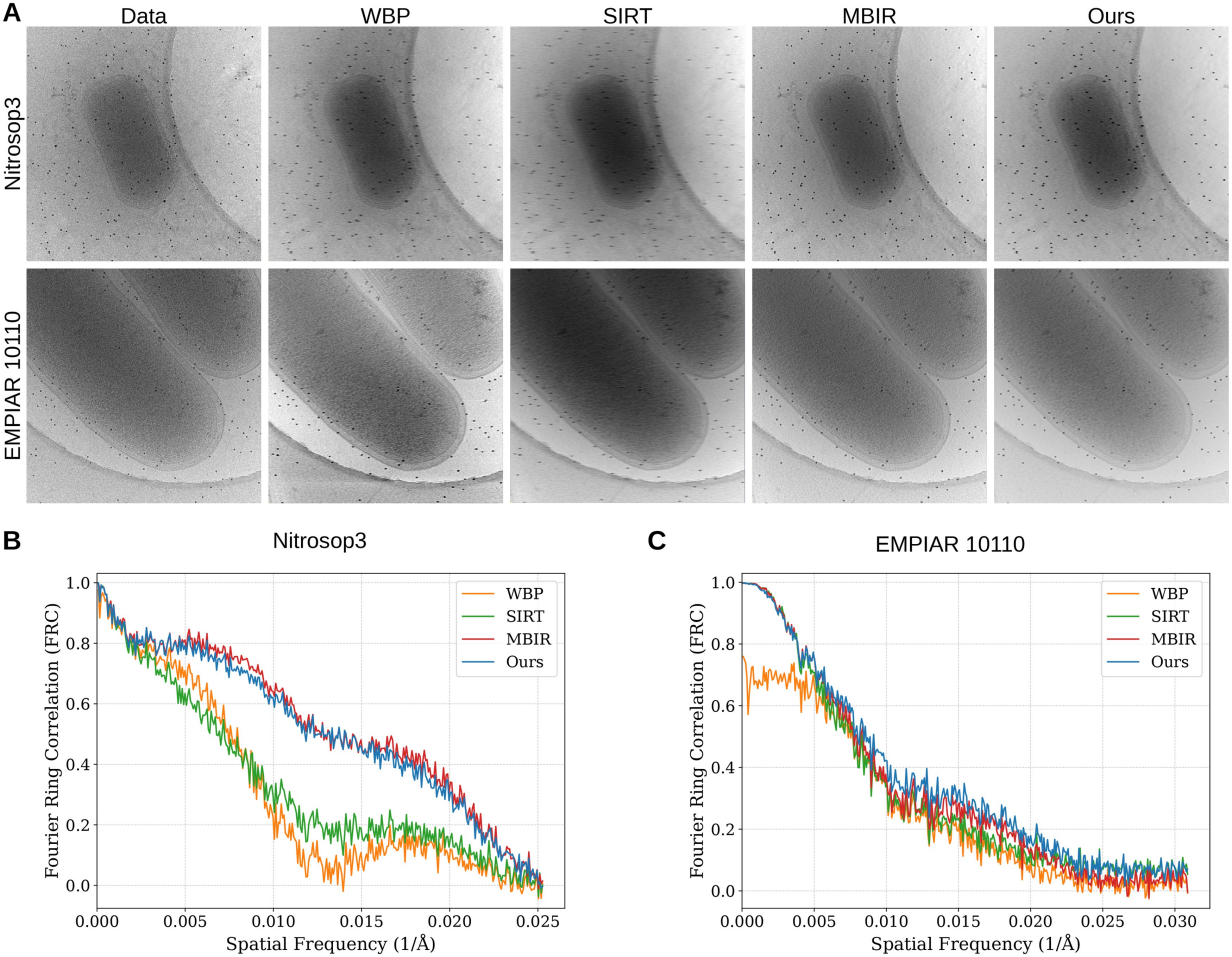
Evaluation of information restoration fidelity on experimental cryo-ET data (Nitrosop3 and EMPIAR 10110). (A) Visual comparison between the original raw projection (Data, left column) excluded from reconstruction and the corresponding re-projections generated by WBP, SIRT, MBIR, and our method at 0° tilt angle. (B-C) Leave-one-out FRC curves calculated between the raw images and re-projections for the two datasets.

Figure 4A compares the held-out raw projection at $0^{{}^{\circ}}$ with re-projections generated from tomograms reconstructed without that view. SIRT is visibly affected by artifacts, whereas WBP, MBIR, and our method appear more similar in overall structural recovery, although WBP exhibits a higher noise background. To quantify these differences, Fig. 4B–C report the leave-one-out FRC curves. WBP and SIRT show lower correlations with a clear frequency-dependent decay, while MBIR and our method consistently maintain higher correlations across a wide frequency range. Although minor dataset-specific variations exist, MBIR and our method closely track each other and provide a substantial improvement over the baselines. Overall, the results suggest that our variational framework achieves information restoration fidelity comparable to the computationally intensive MBIR while remaining consistent with the original measurements.

### 3.4 Particle detection performance via template matching

To evaluate whether improved reconstruction quality translates to better downstream particle localization, we performed template matching using Dynamo Castaño-Díez *et al*. (2012). The dataset contains 400 ground-truth particles. Dynamo outputs a ranked list of candidate peaks; we matched each detected peak to the ground truth using nearest-neighbor association and counted it as a true positive if the Euclidean distance was below 10 pixels (approximately one-sixth of the particle diameter). Based on true positives (TP), false positives (FP), and false negatives (FN), we report Precision, Recall, and F1-score:


$$\begin{array}{c} \text{Precision}=\frac{\text{TP}}{\text{TP}+\text{FP}},\;\text{Recall}=\frac{\text{TP}}{\text{TP}+\text{FN}},\\ F1-\text{score}=2\cdot \frac{\text{Precision}\cdot \text{Recall}}{\text{Precision}+\text{Recall}}. \end{array}$$

Precision quantifies how many selected candidates are correct, Recall measures the fraction of recovered ground-truth particles, and F1-score balances the two.

**Figure 5:**
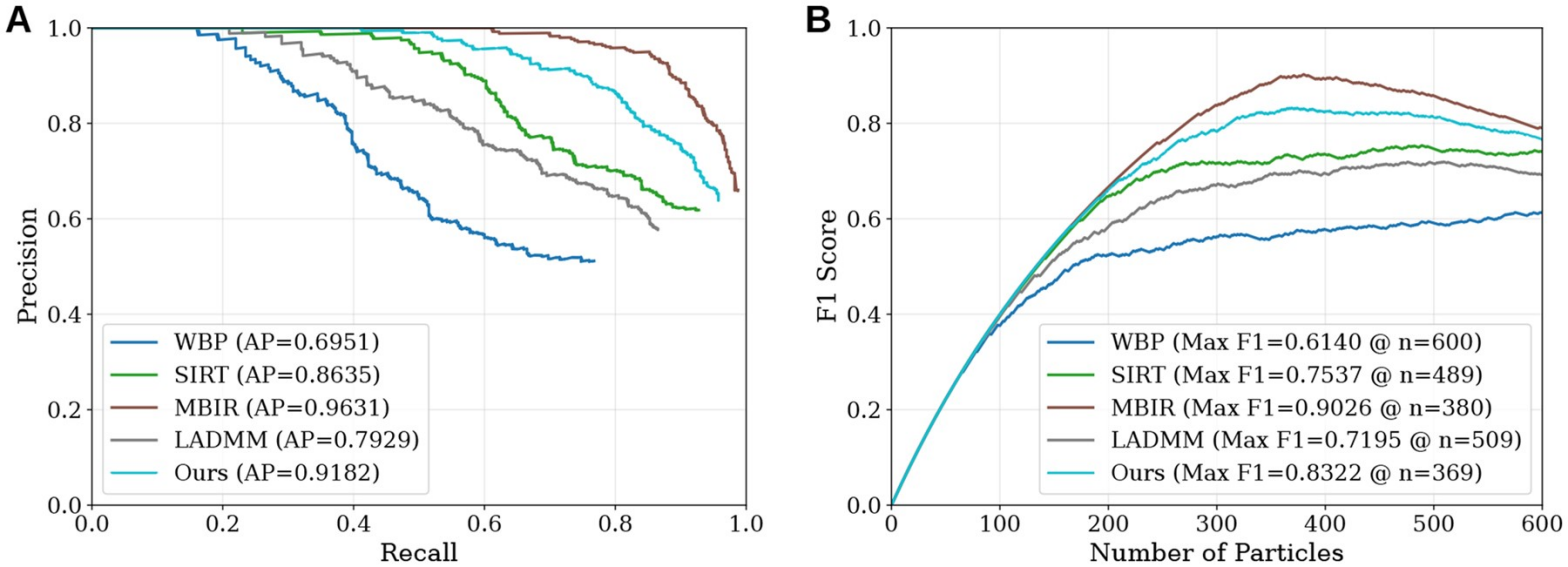
Template matching performance comparison. (A) Precision-Recall curves. The Average Precision (AP) for each method is noted in the legend. (B) F1-score as a function of the number of picked particles. The maximum F1-score and the corresponding number of particles *n* are indicated.

Figure 5 and Table 1 compare the methods in terms of both template matching accuracy and reconstruction efficiency. In terms of detection performance, MBIR achieves the best overall results, with the highest AP and an F1-score of 0.7900. Our method ranks second, reaching an F1-score of 0.7660, which is close to that of MBIR. In addition, our method improves both Precision and Recall compared with WBP, SIRT, and LADMM, indicating that it better preserves structural features relevant for localization. WBP provides the lowest baseline performance with an F1-score of 0.6140, reflecting its sensitivity to noise. SIRT substantially improves the result to 0.7420 by enhancing the visibility of major structures. LADMM achieves an F1-score of 0.6920, which is lower than that of SIRT, suggesting that its regularization may oversuppress or distort discriminative cues needed for template matching, even though the reconstruction appears visually smoother.

**Table 1:** Quantitative comparison of template matching performance and computational efficiency.

Method	TP ↑	FP ↓	FN ↓	Recall ↑	Precision ↑	F1-score ↑	Run time ↓
WBP	307	293	93	0.7675	0.5117	0.6140	**4.3s**
SIRT	371	229	29	0.9275	0.6183	0.7420	17s
MBIR	**395**	**205**	**5**	**0.9875**	**0.6583**	**0.7900**	7h42m
LADMM	346	254	54	0.8650	0.5767	0.6920	22m55s
Ours	383	217	17	0.9575	0.6383	0.7660	1h18m

*Note.* ↑ indicates higher is better, ↓ indicates lower is better. The run time denotes the total time required to reconstruct one tomogram. WBP, SIRT, MBIR, and our method were executed on CPU, whereas LADMM was executed on GPU.

Metrics were calculated based on the top 600 particles picked by Dynamo from a dataset containing 400 ground truth particles. The **best** results are highlighted in **bold**, and the second-best results are underlined.

We further assessed ranking reliability by analyzing the F1-score as a function of the number of picked particles in Fig. 5B. A reliable ranking should peak near the ground-truth count of 400, because the top candidates should contain mostly true particles before false positives accumulate. MBIR peaks at 0.9029 when selecting 380 candidates. Our method shows a similar behavior and peaks at 0.8322 when selecting 369 candidates, which remains close to the ground truth and indicates that true positives are prioritized early in the ranked list. In contrast, LADMM and SIRT peak at substantially larger candidate pools, with 509 and 489 candidates, respectively. This shift implies that more false positives are mixed among higher-ranked detections, so a looser cutoff is needed to recover most true particles.

From the perspective of computational cost, the compared methods show clear differences in reconstruction time. WBP is the fastest method, requiring only 4.3 s to reconstruct one tomogram, followed by SIRT at 17 s. Their short runtimes are mainly due to their simpler reconstruction procedures and the efficient implementation in Tomo3D. WBP consists of projection filtering and a single backprojection step, whereas SIRT, although iterative, uses relatively simple forward- and backprojection-based updates. In contrast, LADMM, our method, and MBIR require 22 min 55 s, 1 h 18 min, and 7 h 42 min per tomogram, respectively, because they involve more complex optimization procedures and generally require more iterative updates, resulting in higher computational cost. These differences in reconstruction speed may be relevant in practical cryo-ET workflows, particularly for large-scale routine reconstruction, dataset screening, and parameter tuning. It should also be noted that LADMM was executed on GPU, whereas WBP, SIRT, MBIR, and our method were executed on CPU. Therefore, these runtimes should be interpreted as practical costs under our implementation settings rather than as a strictly hardware-normalized benchmark.

### 3.5 Resolution assessment via subtomogram averaging

To assess the preservation of high-frequency structural details and the reliability of the reconstructed volumes for downstream analysis, we performed subtomogram averaging (STA) on the simulated dataset. Specifically, 400 subtomograms ($80^{3}$ voxels, voxel size 4 Å) were extracted from the tomograms reconstructed by each method using the ground truth particle coordinates. Crucially, to decouple reconstruction quality from potential alignment inaccuracies, we bypassed the iterative alignment process and utilized the ground truth rotation and translation parameters from the simulation to directly average the subtomograms.

**Figure 6:**
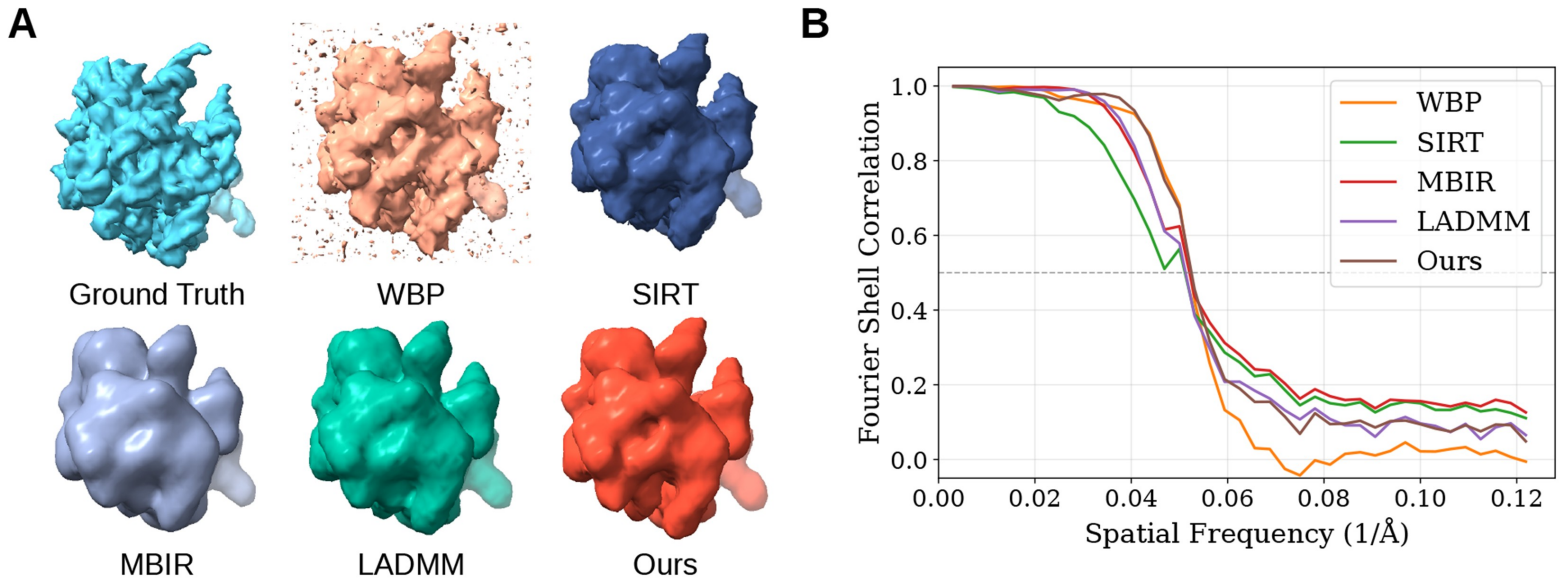
Comparison of subtomogram averaging results. (A) Visual comparison between the reference 5lzf map (Ground Truth) and the averaged maps derived from tomograms reconstructed by different methods. (B) FSC curves calculated between the averaged maps and the ground truth reference. The dashed line indicates the FSC = 0.5 threshold.

Figure 6A displays the resulting averaged maps alongside the reference map. Visual inspection reveals that the averaged map from WBP contains more noticeable noise artifacts than those from the other methods. Conversely, while MBIR and LADMM effectively suppress noise, they tend to over-smooth the volume, resulting in the loss of fine surface textures. In contrast, the reconstructions obtained via SIRT and our method successfully retain sharper structural features.

The fidelity of the resulting density maps was quantified using the Fourier shell correlation (FSC) calculated against the ground truth reference map, as illustrated in Fig. 6B. Adopting the standard 0.5 threshold criterion van Heel and Schatz (2005), our method achieves a resolution of 19.06 Å. This represents a measurable improvement over SIRT (19.55 Å) and LADMM (19.52 Å), while marginally surpassing WBP (19.16 Å) and MBIR (19.22 Å). These quantitative results confirm that our proposed composite regularization strategy successfully balances robust noise suppression with the preservation of high-frequency structural information, effectively avoiding the resolution loss often associated with conventional regularization schemes.

## 4 Conclusion

We proposed a variational framework for 3D cryo-ET reconstruction under low SNR and geometrically incomplete data. The method combines a physically consistent WLS data-fidelity term with composite regularization to correct detector-truncation-induced vignetting without volume padding while preserving multi-scale biological structures. The resulting convex problem admits stability guarantees and is solved efficiently via a PDHG scheme with parallelizable updates. Experiments on simulated and experimental datasets demonstrate consistent improvements over standard baselines in noise suppression, contrast, particle detection, and resolution, while mitigating over-smoothing artifacts; moreover, it achieves quality comparable to computationally intensive model-based approaches at substantially lower computational cost.

Despite these results, several limitations warrant further investigation. The regularization hyperparameters are currently tuned manually across datasets, and future work will investigate automated selection strategies based on statistical noise estimation. In addition, the proximal framework naturally accommodates additional priors, and integrating learned priors such as plug-and-play or RED-style pre-trained denoisers is promising for extremely low-dose regimes, provided that stability and convergence can be maintained or appropriately characterized.

## Supplementary Material

btag206_Supplementary_Data
