# Supplementary material for: A variational framework with composite sparse regularization for cryo-electron tomography reconstruction
Source: Bioinformatics. 2026 May 26;42(6):btag206. doi: 10.1093/bioinformatics/btag206 (PMC13242182; doi:10.1093/bioinformatics/btag206)
Supplement: btag206_Supplementary_Data [file btag206_supplementary_data.pdf]

Supplementary material for "A variational framework with composite sparse regularization for cryo-electron tomography reconstruction"

Chenyun Yu[1,2,†], Zihe Xu[1,†], Qiong Zeng[3], Xiaohua Wan[4], Haythem El-Messiry[5], Fa Zhang[4,*], Renmin Han[1,2,*]

[1]Frontiers Science Center for Nonlinear Expectations (Ministry of Education), Research Center for Mathematics and Interdisciplinary Sciences; Qilu Hospital (Qingdao), Cheeloo College of Medicine, Shandong University, Qingdao 266237, China; [2]College of Medical Information and Engineering, Ningxia Medical University, Yinchuan, 750004, China; [3]School of Computer Science and Technology, Shandong University, Qingdao, 266237, China; [4]School of Medical Technology, Beijing Institute of Technology, Beijing, 100081, China; [5]Canadian University Dubai, Dubai, 117781, United Arab Emirates

S1 Convergence Analysis

To establish convergence of the proposed PDHG algorithm, we cast the reconstruction model into the standard primal–dual form in (Chambolle and Pock, 2011). Let $\boldsymbol{u} \in \mathbb{R}^N$ denote the reconstructed volume, and let $\boldsymbol{K} : \mathbb{R}^N \to \mathbb{R}^{M+3N+Q}$ be a bounded linear operator that stacks the forward projector and the regularization transforms. We consider the convex optimization problem

$$\min_{\boldsymbol{u}} F(\boldsymbol{K}\boldsymbol{u}) + G(\boldsymbol{u}),$$

where $G(\boldsymbol{u}) \equiv 0$. Denote $\boldsymbol{y} = \boldsymbol{K}\boldsymbol{u}$ and use the block decomposition $\boldsymbol{y} = (\boldsymbol{y}_A, \boldsymbol{y}_\nabla, \boldsymbol{y}_\mathcal{C})$. The function F is defined as follows:

$$F(\boldsymbol{y}) = F_A(\boldsymbol{y}_A) + F_\nabla(\boldsymbol{y}_\nabla) + F_\mathcal{C}(\boldsymbol{y}_\mathcal{C}), \tag{1}$$

where

$$F_A(\boldsymbol{y}_A) = \frac{1}{2}\|\boldsymbol{y}_A - \tilde{\boldsymbol{b}}\|^2_{\boldsymbol{R}}$$

is the data-fidelity term, and F_∇ and $F_\mathcal{C}$ are ℓ_1-norm penalties (with weights $\lambda_1 > 0$ and $\lambda_2 > 0$, respectively), consistent with the objective used in our PDHG implementation.

The PDHG algorithm seeks a saddle point $(\boldsymbol{u}^*, \boldsymbol{v}^*)$ of the Lagrangian

$$\mathcal{L}(\boldsymbol{u}, \boldsymbol{v}) = \langle \boldsymbol{K}\boldsymbol{u}, \boldsymbol{v}\rangle - F^*(\boldsymbol{v}) + G(\boldsymbol{u}), \tag{2}$$

where $\boldsymbol{v} = (\boldsymbol{v}_A, \boldsymbol{v}_\nabla, \boldsymbol{v}_\mathcal{C})$ is the dual variable and F^* is the Legendre–Fenchel conjugate of F. The primal and dual problems are

$$\inf_{\boldsymbol{u}} \sup_{\boldsymbol{v}} \mathcal{L}(\boldsymbol{u}, \boldsymbol{v}) = \inf_{\boldsymbol{u}} \{F(\boldsymbol{K}\boldsymbol{u}) + G(\boldsymbol{u})\},$$

and

$$\sup_{\boldsymbol{v}} \inf_{\boldsymbol{u}} \mathcal{L}(\boldsymbol{u}, \boldsymbol{v}) = \sup_{\boldsymbol{v}} \{-F^*(\boldsymbol{v}) - G^*(-\boldsymbol{K}^*\boldsymbol{v})\}.$$

We denote the optimal primal and dual values as p^* and d^*, respectively. Following (Chambolle and Pock, 2011), convergence holds once (i) F^* and G are proper, convex, and lower semicontinuous, and (ii) the set of saddle points of $\mathcal{L}$ is non-empty. We verify these conditions via the following lemmas.

Lemma 1. *The functions F^* and G are proper, convex, and lower semicontinuous (l.s.c.).*

Proof. The function $G(\boldsymbol{u}) \equiv 0$ is trivially proper, convex, and continuous on $\mathbb{R}^N$, which implies lower semicontinuous.

Consider the function $F(\boldsymbol{v})$ defined in Eq. (1). It is composed of a shifted squared Euclidean norm F_A and scaled ℓ_1-norms F_∇ and $F_\mathcal{C}$. Since norms and quadratic functions are continuous and convex, their sum F retains these properties. A fundamental result in convex analysis states that the Legendre-Fenchel conjugate of a proper, convex, and l.s.c. function is itself proper, convex, and l.s.c. (Bauschke and Combettes, 2017, Proposition 13.11). Therefore, F^* satisfies the required conditions. □

Lemma 2. *The set of primal solutions is non-empty, i.e., there exists $\boldsymbol{u}^* \in \arg\min_{\boldsymbol{u}} \{F(\boldsymbol{K}\boldsymbol{u}) + G(\boldsymbol{u})\}$.*

Proof. Let $\Phi(\boldsymbol{u}) = F(\boldsymbol{K}\boldsymbol{u}) + G(\boldsymbol{u}) = F(\boldsymbol{K}\boldsymbol{u})$. Since F and $\boldsymbol{K}$ are continuous, the composition Φ is l.s.c. Furthermore, Φ is proper because $\Phi(\boldsymbol{0}) = \frac{1}{2}\|\boldsymbol{R}^{1/2}\tilde{\boldsymbol{b}}\|_2^2 < \infty$. According to (Bauschke and Combettes, 2017, Corollary 11.16), the existence of a minimizer is guaranteed if Φ is coercive, meaning $\Phi(\boldsymbol{u}) \to \infty$ as $\|\boldsymbol{u}\| \to \infty$.

The composite objective includes the term $\lambda_2\|\mathcal{C}\boldsymbol{u}\|_1$. The curvelet transform $\mathcal{C}$ constitutes a tight frame, implying that $\mathcal{C}$ is an injective linear operator with a trivial kernel, that is $\ker(\mathcal{C}) = \{\boldsymbol{0}\}$. Consequently, the mapping $\boldsymbol{u} \mapsto \|\mathcal{C}\boldsymbol{u}\|_1$ defines a norm on the finite-dimensional space $\mathbb{R}^N$. Due to the equivalence of norms, there exists a constant $c > 0$ such that $\|\mathcal{C}\boldsymbol{u}\|_1 \geq c\|\boldsymbol{u}\|_2$.

Thus, $\|\boldsymbol{u}\| \to \infty$ implies $\|\mathcal{C}\boldsymbol{u}\|_1 \to \infty$. Since all other terms in F are non-negative, it follows that $\Phi(\boldsymbol{u}) \to \infty$. The coercivity condition is met, ensuring the existence of a primal minimizer $\boldsymbol{u}^*$. □

Lemma 3. *Strong duality $p^* = d^*$ holds, and the set of dual solutions is non-empty, i.e., there exists $\boldsymbol{v}^* \in \arg\max_{\boldsymbol{v}} \{-F^*(\boldsymbol{v}) - G^*(-\boldsymbol{K}^*\boldsymbol{v})\}$.*

Proof. We verify Slater's constraint qualification condition

$$\boldsymbol{0} \in \operatorname{int}(\operatorname{dom}(F) - \boldsymbol{K}\operatorname{dom}(G)).$$

The effective domain of G is $\operatorname{dom}(G) = \mathbb{R}^N$. Based on Eq. (1), F is finite everywhere, so $\operatorname{dom}(F) = \mathbb{R}^{M+3N+Q}$. The condition simplifies to $\boldsymbol{0} \in \mathbb{R}^{M+3N+Q}$, which is trivially satisfied.

Since the primal optimal value p^* is finite as established in Lemma 2, and Slater's condition holds, the Fenchel Duality Theorem (Bauschke and Combettes, 2017, Theorem 15.23) guarantees that strong duality $p^* = d^*$ holds and that the dual supremum is attained by some $\boldsymbol{v}^*$. □

Theorem 4. *Let the primal and dual step sizes $\tau, \sigma > 0$ satisfy the condition $\tau\sigma\|\boldsymbol{K}\|^2 < 1$, and let the extrapolation parameter $\theta = 1$. Then, the sequence $(\boldsymbol{u}^k, \boldsymbol{v}^k)$ generated by the PDHG algorithm converges to a saddle point $(\boldsymbol{u}^*, \boldsymbol{v}^*)$ of the Lagrangian $\mathcal{L}$.*

Proof. Based on (Chambolle and Pock, 2011, Theorem 1), convergence is guaranteed if two conditions are met: (1) the functions G and F^* are proper, convex, and l.s.c., and (2) the set of saddle points for $\mathcal{L}(\boldsymbol{u}, \boldsymbol{v})$ is non-empty.

Lemma 1 satisfies the first condition. For the second condition, standard convex analysis results (Bauschke and Combettes, 2017, Corollary 19.19) state that a pair $(\boldsymbol{u}^*, \boldsymbol{v}^*)$ is a saddle point if and only if $\boldsymbol{u}^*$ is a primal minimizer, $\boldsymbol{v}^*$ is a dual maximizer, and strong duality holds. Lemmas 2 and 3 confirm that such primal and dual solutions exist and that strong duality holds. Thus, the set of saddle points is non-empty.

Consequently, the algorithm converges for any choice of step sizes satisfying the bounded operator norm condition. □

S2 Datasets and Experimental Setup

To comprehensively validate the performance of our proposed framework, we conducted extensive experiments on one simulated dataset and four real-world cryo-ET datasets. These datasets cover a diverse range of biological specimens, from purified macromolecular assemblies to complex *in situ* cellular environments. For all real-world experiments, the raw projection images were spatially binned by a factor of 4 prior to reconstruction.

Table S1: Dataset statistics for the real and simulated cryo-ET datasets used in our experiments.

Dataset	Tilt Range	# Proj.	Raw Image Size (px)	Px Size (Å)	Binning	Thickness (px)
EMPIAR-10643	$(-60^\circ, 60^\circ, 3^\circ)$	41	4096 × 4096	1.179	4	300
EMPIAR-10499	$(-60^\circ, 60^\circ, 3^\circ)$	41	3838 × 3710	1.7005	4	400
Nitrosop3	$(-55^\circ, 55^\circ, 1^\circ)$	111	3838 × 3710	4.90	4	300
EMPIAR-10110	$(-60^\circ, 60^\circ, 1^\circ)$	121	3838 × 3710	4.04	4	300
Simulated Dataset	$(-60^\circ, 60^\circ, 2^\circ)$	61	1024 × 1024	4.00	1	400

Note: The tilt range format is denoted as (min, max, increment). # Proj. denotes the number of projections.

The first real-world dataset, EMPIAR-10643 (Iudin et al., 2016; Ni et al., 2022), comprises a tilt series of HIV-1 GagdeltaMASP1T8I assemblies. The data were acquired using a 300 kV Caltech Polara microscope equipped with a Gatan K2 Summit direct electron detector. The tilt series contains 41 projections spanning a

range of $-60°$ to $60°$ with $3°$ increments, collected at an average dosage of $205\,e^- \cdot Å^{-2}$. The raw projection images have dimensions of 4096×4096 pixels with a pixel size of 1.179 Å. The data can be accessed at https://www.ebi.ac.uk/empiar/EMPIAR-10643/.

The second dataset, EMPIAR-10499, features *in situ* tilt series of *Mycoplasma pneumoniae* cells treated with chloramphenicol (Tegunov et al., 2021). The data were acquired using a 300 kV Titan Krios microscope equipped with a Gatan K2 Summit direct electron detector and a Quantum energy filter. The tilt series were collected using a dose-symmetric scheme spanning a tilt range of $\pm 60°$ with $3°$ increments, comprising a total of 41 projections with a cumulative dose of approximately $120\,e^- \cdot Å^{-2}$. The projection images have dimensions of 3838×3710 pixels, corresponding to a calibrated pixel size of 1.7005 Å. The data can be accessed at https://www.ebi.ac.uk/empiar/EMPIAR-10499/.

The third dataset, Nitrosop3, sourced from the ETDB-Caltech database (Qin et al., 2017; Ortega et al., 2019), represents the marine archaeon *Nitrosopumilus maritimus*. The tilt series was collected on an FEI Tecnai Polara microscope at 300 kV with a Gatan K2 camera. It consists of 111 projections spanning $-55°$ to $55°$ at $1°$ increments, with a total dosage of approximately $180\,e^- \cdot Å^{-2}$. The images measure 3838×3710 pixels with a pixel size of 4.9 Å. The data can be accessed at https://doi.org/10.5281/zenodo.19366744.

The fourth dataset, EMPIAR-10110, captures intact *Vibrio cholerae* O395 N1 cells and was originally acquired to investigate the toxin-coregulated pilus machine (Chang et al., 2017). Data collection was performed on a 300 kV FEI Tecnai Polara microscope equipped with a Gatan K2 Summit direct electron detector and a Gatan energy filter. The tilt series comprises 121 projections acquired from $-60°$ to $60°$ with $1°$ increments, with a total cumulative dose of approximately $160\,e^- \cdot Å^{-2}$. The raw images are 3838×3710 pixels with a pixel size of 4.04 Å. The data can be accessed at https://www.ebi.ac.uk/empiar/EMPIAR-10110/.

In addition to the real-world data, we generated a simulated dataset to provide a ground truth for quantitative metrics such as template matching and resolution assessment. We utilized the atomic model of the human precatalytic spliceosome (PDB ID: 5lzf) to generate 400 macromolecular complexes randomly distributed within a volume. The simulated projections were generated with a tilt range of $\pm 60°$ at $2°$ increments, with dimensions of 1024×1024 pixels and a pixel size of 4.0 Å. The data can be accessed at https://doi.org/10.5281/zenodo.19367038.

S3 Parameter Sensitivity Analysis for WBP and SIRT in Tomo3D

To further assess the baseline settings used in the main experiments, we examined the influence of key parameters in the Tomo3D implementations of WBP and SIRT on representative datasets. For WBP, we compared two Hamming weighting filter frequencies, namely 0.0 and 0.35. For SIRT, we evaluated four iteration numbers, namely 5, 10, 20, and 30. The corresponding visual comparisons are shown in Figure S1.

For WBP, the reconstruction obtained with a Hamming weighting filter frequency of 0.0 showed slightly better structural preservation and more favorable signal-to-noise characteristics than that obtained with

0.35. For SIRT, the reconstruction with 5 iterations exhibited relatively high contrast but remained overly smooth, suggesting insufficient structural recovery. Increasing the number of iterations to 20 and 30 revealed more structural details, but also introduced more noticeable noise. Among the tested settings, 10 iterations provided the best balance between structural detail preservation and noise suppression. Based on these observations, we used a Hamming weighting filter frequency of 0.0 for WBP and 10 iterations for SIRT in all experiments reported in the main text.

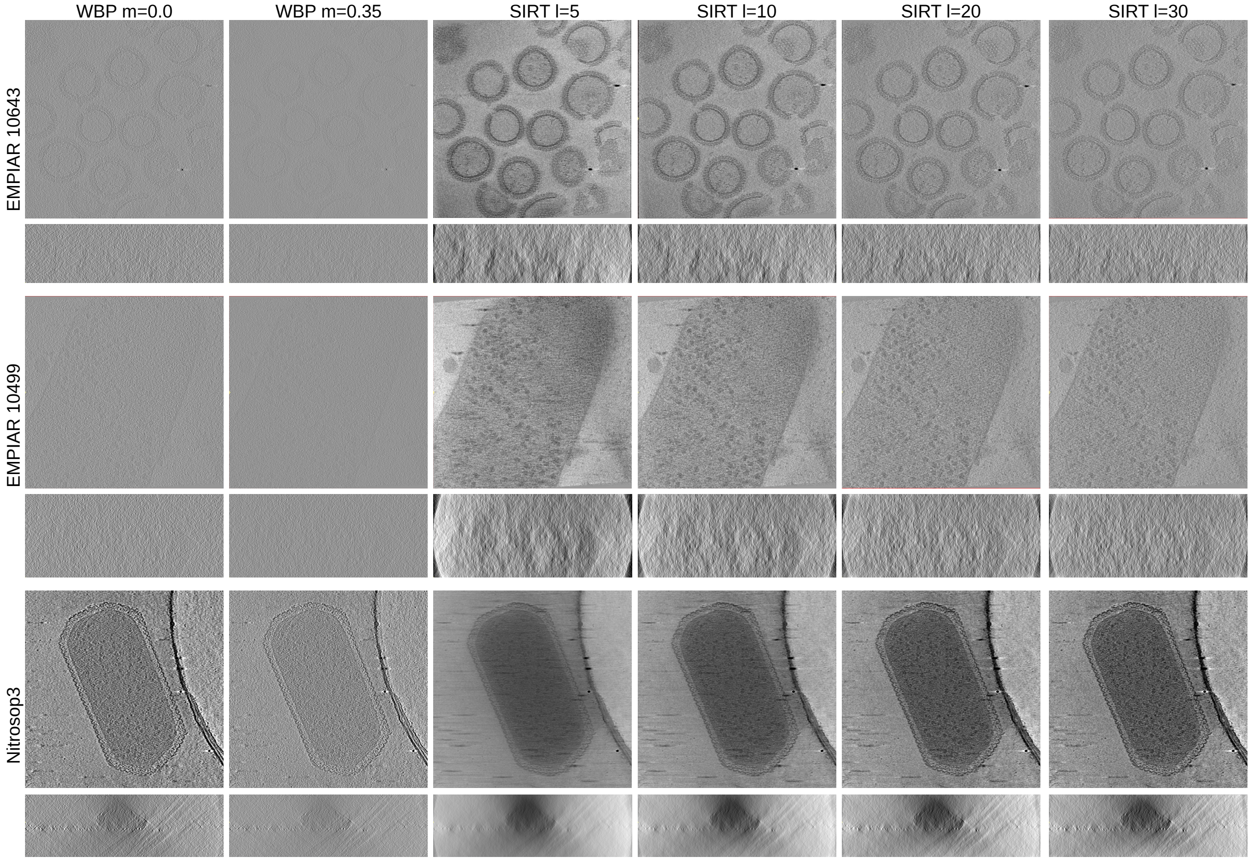

Figure S1: Visual comparison of WBP and SIRT in Tomo3D reconstructions with different parameter settings. For WBP, the Hamming weighting filter frequencies of 0.0 and 0.35 are compared. For SIRT, the number of iterations (5, 10, 20, and 30) are compared. The figure includes both XY and XZ slices to illustrate the effects of these parameters on visual quality and artifact suppression.

References

Bauschke, H. H. and P. L. Combettes (2017). *Convex Analysis and Monotone Operator Theory in Hilbert Spaces*. Springer International Publishing.

Chambolle, A. and T. Pock (2011). A first-order primal-dual algorithm for convex problems with applications to imaging. *Journal of Mathematical Imaging and Vision 40*(1), 120–145.

Chang, Y.-W., A. Kjær, D. R. Ortega, G. Kovacikova, J. A. Sutherland, L. A. Rettberg, R. K. Taylor, and G. J. Jensen (2017). Architecture of the vibrio cholerae toxin-coregulated pilus machine revealed by electron cryotomography. *Nature Microbiology 2*(4).

Iudin, A., P. K. Korir, J. Salavert-Torres, G. J. Kleywegt, and A. Patwardhan (2016, March). Empiar: a public archive for raw electron microscopy image data. *Nature Methods 13*(5), 387–388.

Ni, T., T. Frosio, L. Mendonça, Y. Sheng, D. Clare, B. A. Himes, and P. Zhang (2022, January). High-resolution in situ structure determination by cryo-electron tomography and subtomogram averaging using emclarity. *Nature Protocols 17*(2), 421–444.

Ortega, D. R., C. M. Oikonomou, H. J. Ding, P. Rees-Lee, and G. J. Jensen (2019, April). Etdb-caltech: A blockchain-based distributed public database for electron tomography. *PLOS ONE 14*(4), e0215531.

Qin, W., K. R. Heal, R. Ramdasi, J. N. Kobelt, W. Martens-Habbena, A. D. Bertagnolli, S. A. Amin, C. B. Walker, H. Urakawa, M. Könneke, et al. (2017). Nitrosopumilus maritimus gen. nov., sp. nov., nitrosopumilus cobalaminigenes sp. nov., nitrosopumilus oxyclinae sp. nov., and nitrosopumilus ureiphilus sp. nov., four marine ammonia-oxidizing archaea of the phylum thaumarchaeota. *International journal of systematic and evolutionary microbiology 67*(12), 5067–5079.

Tegunov, D., L. Xue, C. Dienemann, P. Cramer, and J. Mahamid (2021, February). Multi-particle cryo-em refinement with m visualizes ribosome-antibiotic complex at 3.5 Å in cells. *Nature Methods 18*(2), 186–193.
